# *Ureaplasma* Species Differentially Modulate Pro- and Anti-Inflammatory Cytokine Responses in Newborn and Adult Human Monocytes Pushing the State Toward Pro-Inflammation

**DOI:** 10.3389/fcimb.2017.00484

**Published:** 2017-11-28

**Authors:** Kirsten Glaser, Christine Silwedel, Markus Fehrholz, Ana M. Waaga-Gasser, Birgit Henrich, Heike Claus, Christian P. Speer

**Affiliations:** ^1^University Children's Hospital, University of Wuerzburg, Wuerzburg, Germany; ^2^Department of Surgery I, Molecular Oncology and Immunology, University of Wuerzburg, Wuerzburg, Germany; ^3^Institute of Medical Microbiology and Hospital Hygiene, University Clinic of Heinrich-Heine University Duesseldorf, Duesseldorf, Germany; ^4^Institute for Hygiene and Microbiology, University of Wuerzburg, Wuerzburg, Germany

**Keywords:** *Ureaplasma*, infection, inflammation, immunomodulation, chorioamnionitis, neonatal morbidity, monocytes, cord blood

## Abstract

**Background:** *Ureaplasma* species have been associated with chorioamnionitis and preterm birth and have been implicated in the pathogenesis of neonatal short and long-term morbidity. However, being mostly commensal bacteria, controversy remains on the pro-inflammatory capacity of *Ureaplasma*. Discussions are ongoing on the incidence and impact of prenatal, perinatal, and postnatal infection. The present study addressed the impact of *Ureaplasma* isolates on monocyte-driven inflammation.

**Methods:** Cord blood monocytes of term neonates and adult monocytes, either native or LPS-primed, were cultured with *Ureaplasma urealyticum* (*U. urealyticum*) serovar 8 (Uu8) and *Ureaplasma parvum* serovar 3 (Up3). Using qRT-PCR, cytokine flow cytometry, and multi-analyte immunoassay, we assessed mRNA and protein expression of tumor necrosis factor (TNF)-α, interleukin (IL)-1β, IL-8, IL-12p40, IL-10, and IL-1 receptor antagonist (IL-1ra) as well as Toll-like receptor (TLR) 2 and TLR4.

**Results:** Uu8 and Up3 induced mRNA expression and protein release of TNF-α, IL-1β and IL-8 in term neonatal and adult monocytes (*p* < 0.01 and *p* < 0.05). Intracellular protein expression of TNF-α, IL-1β and IL-8 in *Ureaplasma*-stimulated cells paralleled those results. *Ureaplasma*-induced cytokine levels did not significantly differ from LPS-mediated levels except for lower intracellular IL-1β in adult monocytes (Uu8: *p* < 0.05). Remarkably, ureaplasmas did not induce IL-12p40 response and promoted lower amounts of anti-inflammatory IL-10 and IL-1ra than LPS, provoking a cytokine imbalance more in favor of pro-inflammation (IL-1β/IL-10, IL-8/IL-10 and IL-8/IL-1ra: *p* < 0.01, vs. LPS). In contrast to LPS, both isolates induced TLR2 mRNA in neonatal and adult cells (*p* < 0.001 and *p* < 0.05) and suppressed TLR4 mRNA in adult monocytes (*p* < 0.05). Upon co-stimulation, Uu8 and Up3 inhibited LPS-induced intracellular IL-1β (*p* < 0.001 and *p* < 0.05) and IL-8 in adult monocytes (*p* < 0.01), while LPS-induced neonatal cytokines were maintained or aggravated (*p* < 0.05).

**Conclusion:** Our data demonstrate a considerable pro-inflammatory capacity of *Ureaplasma* isolates in human monocytes. Stimulating pro-inflammatory cytokine responses while hardly inducing immunomodulatory and anti-inflammatory cytokines, ureaplasmas might push monocyte immune responses toward pro-inflammation. Inhibition of LPS-induced cytokines in adult monocytes in contrast to sustained inflammation in term neonatal monocytes indicates a differential modulation of host immune responses to a second stimulus. Modification of TLR2 and TLR4 expression may shape host susceptibility to inflammation.

## Introduction

*Ureaplasma urealyticum* (*U. urealyticum*) (serovars 2, 4, 5, 7–13) and the separate species *Ureaplasma parvum* (serovars 1, 3, 6, 14) are generally regarded as commensal bacteria being detected in the lower genital tract of 40–80% of women of reproductive age (Abele-Horn et al., 1997; Volgmann et al., 2005; Hunjak et al., 2014). In pregnant women, however, upper genital tract infection with *Ureaplasma* species (spp.) has been associated with chorioamnionitis (CA), adverse pregnancy outcome and preterm birth (i.e., delivery <37 weeks of gestation), especially at gestational ages <30 weeks (Digiulio et al., 2008; Cox et al., 2016; Sweeney et al., 2017). Even in moderate preterm infants (i.e., birth between 32 and 36 weeks of gestation) *Ureaplasma* spp. are one of the most commonly recovered organisms in case of histologically confirmed CA (Kasper et al., 2010; Namba et al., 2010; Sweeney et al., 2016). With upper genital tract infections often being polymicrobial and with genital tract colonization occurring both among mothers with preterm birth and among mothers with full-term delivery, nevertheless, the role of *Ureaplasma* in disease manifestation in pregnancy remains controversial (Kafetzis et al., 2004; Kirchner et al., 2007; Digiulio et al., 2008; Donders et al., 2017).

In preterm and term neonates, *Ureaplasma* spp. have been described as pathogens of invasive diseases, such as pneumonia, sepsis, and meningitis (Waites et al., 2005; Viscardi, 2014; Glaser and Speer, 2015). Moreover, epidemiologic and experimental studies indicate an association of prenatal and perinatal *Ureaplasma* infection with fetal inflammatory response and neonatal short and long-term morbidity (Viscardi et al., 2004, 2008; Kirchner et al., 2007; Berger et al., 2009; Lowe et al., 2014). *Ureaplasma* spp. have been reported to be the most common organisms isolated from the amniotic fluid, cord blood, respiratory tract and the cerebrospinal fluid of preterm infants who develop bronchopulmonary dysplasia (BPD) (Goldenberg et al., 2008; Sung et al., 2011; Lowe et al., 2014; Viscardi, 2014). However, while respiratory tract colonization has been associated with BPD by several investigations (Schelonka et al., 2005; Viscardi and Hasday, 2009; Lowe et al., 2014), multivariable analysis did not confirm this relationship in other studies (Van Waarde et al., 1997; Payne et al., 2010, 2012; Kacerovsky et al., 2014).

Vertical transmission rates of *Ureaplasma* spp. in pregnancy are highly variable, ranging from 15 to 88%, and seem to be inversely related to gestational age (Sánchez and Regan, 1990; Alfa et al., 1995; Chua et al., 1999; Kafetzis et al., 2004). Detection rates in initial tracheal aspirates or nasopharyngeal secretions range from 10 to 50% in term newborns (Chua et al., 1999; Kafetzis et al., 2004; Waites et al., 2005) and 24–52% in preterm infants <32 weeks (Chua et al., 1999; Kafetzis et al., 2004; Goldenberg et al., 2008; Viscardi and Hasday, 2009; Sung et al., 2011; Payne et al., 2012). Preterm colonization seems to be inversely proportional to gestational age, with 65% of preterm infants <26 weeks being colonized vs. 31% of preterm infants with a gestational age between 26 and 32 weeks (Sung et al., 2011). Of note, in preterm infants <32 weeks of gestation, up to 23% of infants may have positive blood cultures for *Ureaplasma* spp. and positive PCR results in venous blood and/or cerebrospinal fluid samples (Goldenberg et al., 2008; Viscardi et al., 2008). The clinical relevance of detecting *Ureaplasma* spp. in microbiological specimen remains subject of discussion. For reasons of low pathogenicity in children and adults and the very common isolation from genitourinary samples, controversy remains concerning the impact of *Ureaplasma* colonization on infection and inflammation-related morbidities (Volgmann et al., 2005; Waites et al., 2005; Viscardi, 2014; Glaser and Speer, 2015; Sweeney et al., 2017).

In pregnancy, intrauterine detection of *Ureaplasma* spp. has been linked to choriodecidual and amniotic inflammation (Aaltonen et al., 2007; Kasper et al., 2010; Namba et al., 2010; Oh et al., 2010; Kacerovsky et al., 2012). Invasive diseases in preterm and term neonates have been associated with increased peripheral leukocyte counts, increased numbers of neutrophils in airways secretions, and characteristic changes in cerebrospinal fluid profiles (Waites et al., 2005; Glaser and Speer, 2015). Clinical studies in preterm infants suggest an association of *Ureaplasma* respiratory tract colonization with bronchopulmonary inflammation and altered lung development (Groneck et al., 1996; Patterson et al., 1998; Viscardi et al., 2006). As far as systemic inflammation is concerned, some studies in preterm infants indicate inflammatory cytokine responses upon neonatal *Ureaplasma* infection (Goldenberg et al., 2008; Hassanein et al., 2012; Viscardi, 2014), while others failed to correlate detection of *Ureaplasma* spp. with systemic inflammation (Payne et al., 2010, 2012; Kacerovsky et al., 2014).

*In vitro* data on *Ureaplasma*-induced inflammation are limited. This study was designed to expand current knowledge of *Ureaplasma*-induced immune responses in human monocytes. Monocytes are a well-known source of early cytokine responses, comprising pro-inflammatory tumor necrosis factor (TNF)-α, interleukin (IL)-1β and IL-8 (Sims and Smith, 2010; Arango Duque and Descoteaux, 2014), immunoregulatory IL-12 as well as anti-inflammatory IL-10 and IL-1 receptor antagonist (IL-1ra) (Trinchieri, 2003; Sabat et al., 2010; Sims and Smith, 2010). Pathogen-induced release of these mediators is triggered by pattern recognition receptors, such as Toll-like receptors (TLRs) (Kawai and Akira, 2010). Providing innate immune response against the most common pathogens involved in CA and neonatal infection, TLR2 and TLR4 may play a particular role in pathogen-induced intrauterine and neonatal inflammation (Kawasaki and Kawai, 2014). Using qRT-PCR, cytokine flow cytometry and multi-analyte immunoassay, we assessed the expression of the given cytokines as well as TLR2 and TLR4 in term neonatal and adult human monocytes stimulated with viable *U. urealyticum* and *U. parvum* in the absence or presence of *Escherichia coli* (*E. coli*) lipopolysaccharide (LPS).

## Materials and methods

### Bacterial strains and culture conditions

Two *Ureaplasma* strains were used, both obtained from the American Tissue Culture Collection (ATCC) (European distributor LGC Standards GmbH, Wesel, Germany): ATCC *U. urealyticum* strain of serovar 8 (Uu8) (ATCC 27618) and ATCC *U. parvum* strain of serovar 3 (Up3) (ATCC 27815), both often associated with disease manifestation (Waites et al., 2009; Xiao et al., 2011). Frozen stocks of 0.5 ml aliquots were prepared from mid-logarithmic-phase broth culture and stored at −80°C until use. For each experiment, both isolates were inoculated 1: 10 in 5 ml in-house medium (“broth”), containing 82% autoclaved PPLO medium (Becton, Dickinson & Company, Franklin Lakes, NJ, USA), 10% heat-inactivated horse serum (v/v), 7% urea (2% aqueous solution) and 1% phenol red (2%) (each obtained from Sigma-Aldrich, St. Louis, CA, USA), passage through a 0.2 micron filter membrane and adjusted to pH 6.5. 10-fold serial dilutions of both strains were incubated overnight to obtain titers of 5 × 10^8^ color-changing units (CCU)/ml of viable organisms. Determination of CCUs was performed in 96-well plates (Greiner, Frickenhausen, Germany) by 10-fold serial dilutions in 200 μl broth as described earlier (Taylor-Robinson et al., 1966). The number of CCUs was determined in duplicate. Corresponding amount of Uu8 and Up3 DNA in copy numbers (cn) was determined at the Institute of Medical Microbiology and Hospital Hygiene Duesseldorf, Germany (Mobius et al., 2012). Viability of inoculated organisms was confirmed by control cultures in “broth” and on selective agar plates (medco Diagnostika GmbH, Ottobrunn, Germany).

### Enrichment of CD14^+^ monocytes from cord blood and peripheral blood mononuclear cells

Umbilical cord blood samples were taken from healthy term newborns delivered by elective cesarean section. Infants were excluded if (i) clinical or laboratory evidence of CA was present, (ii) congenital infection was suspected or confirmed or (iii) congenital malformation had been diagnosed. Written informed consent had been obtained from both parents the day before. The study was conducted in accordance with the World Medical Association Declaration of Helsinki and had been approved by the Ethic Committee of the Medical Faculty of Wuerzburg. Cord blood was collected from the umbilical vein by using a closed collecting system (Maco Pharma International GmbH, Tourcouing, France), and was processed within 2 h. Adult CD14^+^ cells were isolated from randomized leukocyte concentrates obtained from apheresis products of healthy adult donors at the Department of Immunohematology and Transfusion Medicine, University Hospital Wuerzburg. Due to randomization and anonymization donors' individual consent was not required. Cord blood and peripheral blood mononuclear cells (PBMCs) were isolated by Ficoll-Paque gradient centrifugation (Linaris GmbH, Dossenheim, Germany) with cord blood being diluted 1: 3 with NaCl 0.9%. Neonatal and adult CD14^+^ monocytes were enriched by magnetic-activated cell sorting using CD14 MicroBeads® (Miltenyi Biotec GmbH, Bergisch Gladbach, Germany) and re-suspended in RPMI 1640 medium (Sigma-Aldrich) containing 10% fetal bovine serum (Thermo Fisher Scientific, Darmstadt, Germany). CD14^+^ purity was >90% as determined by flow cytometry.

### Cell culture and stimulation assays

Monocytes obtained from *n* = 6 cord blood donors and *n* = 6 adult donors were transferred to culture plates (Greiner) without pooling and seeded at a density of 1 × 10^6^ cells/well. Cells rested for 2 h. Uu8 and Up3 suspensions were added at a concentration of 10^8^ CCU/ml viable organisms, which corresponded to 200 μl per well and 1.3 × 10^6^−1.8 × 10^7^ cn/ml of Uu8 and Up3. Amounts of ureaplasmas recovered from the cervical fluid of pregnancies complicated by preterm labor <37 weeks range from 5 × 10^6^ to 1.5 × 10^8^ cn/ml and *Ureaplasma* load in the amniotic fluid of preterm births <37 weeks of gestation ranges from 4 × 10^3^ to 5.2 × 10^7^ cn/ml (Kacerovsky et al., 2014; Musilova et al., 2016), indicating that the concentrations applied in our study correspond to levels *in vivo*. Monocytes were stimulated at 37°C in a humidified atmosphere with 5% CO_2_. For studies on LPS-primed monocytes, LPS from *E. coli* serotype 055:B5 (Sigma-Aldrich) was added 90 min prior to infection with ureaplasmas. LPS dose and concentration of CCUs had been determined by preliminary dose-response experiments using 1 ng/ml, 10 ng/ml, 100 ng/ml and 1,000 ng/ml LPS and *Ureaplasma* concentrations of 1 × 10^6^, 1 × 10^7^, and 1 × 10^8^ CCU/ml according to previous *in vitro* approaches (Li et al., 2002b; Viscardi et al., 2006; Kallapur et al., 2011). Concentrations applied in this study each produced near-maximal cytokine release (Supplemental Figure 1). With regard to cytokine kinetics, preliminary experiments had addressed different incubation periods (2, 4, 8, 14, and 40 h). For the majority of cytokines analyzed, mRNA expression had peaked at 4 h, detection of intracellularly accumulated cytokines had peaked at 14 h incubation and analysis of secreted proteins at 24 h. Cell viability was confirmed >95% after 4, 14, and 24 h of cell culture for native, LPS-primed and *Ureaplasma*-stimulated monocytes. Addition of Brefeldin A (10 μg/ml, Sigma-Aldrich) allowed for intracellular cytokine flow cytometry.

### RNA extraction and quantitative Real-Time reverse transcription polymerase chain reaction (qRT-PCR)

For RNA extraction, monocytes were harvested after 4 h incubation and separated by centrifugation. Total RNA was extracted using NucleoSpin® RNA Kit (Macherey-Nagel, Dueren, Germany) according to the manufacturer's protocol, eluted in 60 μl nuclease-free H_2_O (Sigma-Aldrich) and stored at −80°C until reverse transcription. 0.11–0.52 μg of total RNA of cord blood monocytes and 0.13–0.50 μg of total RNA of adult monocytes was reverse transcribed using High Capacity cDNA Reverse Transcription Kit (Applied Biosystems, ThermoFisher, Carlsbad, CA, USA). The reaction was terminated by heating at 70°C for 10 min. First strand cDNA was stored at −80°C until further processing. For quantitative detection of TNF-α, IL-1β, IL-8, IL-12p40, IL-10, IL-1ra, TLR2, and TLR4 mRNA, cDNA was diluted 1: 10 in deionized, nuclease-free H_2_O and analyzed in duplicates of 25 μl using 12.5 μl iTaq™ Universal SYBR Green Supermix (Bio-Rad Laboratories, Hercules, CA, USA). Primers used for qRT-PCR are given in Table 1. Analysis was performed using a 7,500 Real-Time PCR System (Applied Biosystems). Amplification was normalized to the reference gene *PPIA* (*peptidyl prolyl isomerase A*). Mean fold changes in mRNA expression were calculated using the ΔΔC_T_ method (Livak and Schmittgen, 2001).

**Table 1:** List of primer sequences used for qRT-PCR.

**Gene symbol**	**Sequence accession**	**Forward primer**	**Reverse primer**
*IL18*	NM_000576	TTCATTGCTCAAGTGTCTG	GCACTTCATCTGTTTAGGG
*ILB*	NM_000584	CAGTGCATAAAGACATACTCC	TTTATGAATTCTCAGCCCTC
*IL12p40*	NM_002187	GGACATCATCAAACCTGAC	AGGGAGAAGTAGGAATGTG
*IL10*	NM_000572	GCTGTCATCGATTTCTTCC	GTCAAACTCACTCATGGCT
*IL1RA*	NM_173842	CTTCTATCTGAGGAACAACCA	AGTGATGTTAACTGCCTCC
*PPIA*	NM_021 130	CAGGGTTTATGTGTCAGGG	CCATCCAACCACTCAGTC
*TLR2*	NM_003264	CCAAAGGAGACCTATAGTGAC	GCTTCAACCCACAACTACC
*TLR4*	NM_1 38554	TTATCCAGGTGTGAAATCCA	GATTTGTCTCCACAGCCA
*TNF*	NM_000594	CAGCCTCTTCTCCTTCCT	GGGTTTGCTACAACATGG

### Flow cytometry

For flow cytometry analysis, monocytes were harvested after 14 h of stimulation, transferred into 96-well plates, separated by centrifugation and stained with fixable viability dye (eBioScience, San Diego, CA, USA) and directly conjugated antibodies to surface CD14, CD16, TLR2, and TLR4 (BioLegend, San Diego, CA, USA). Cells were separated by centrifugation again, washed in Phosphate Buffered Saline (PBS) (Sigma-Aldrich) containing 1% human serum (HS) (Biochrom GmbH, Berlin, Germany) and fixed using fixation buffer (BioLegend). Centrifugation and permeabilization in ice-cold methanol (Sigma-Aldrich) was followed by staining with directly conjugated monoclonal antibodies to TNF-α, IL-1β, IL-8, and IL-10 (eBioScience). Monocytes were finally washed and re-suspended in PBS/1 % HS. All specimens were analyzed using a FACSCanto™ II flow cytometer (BD Biosciences, San Jose, CA, USA). A minimum of 10,000 CD14^+^ monocyte-gated events were acquired in list mode and analyzed with FACSDiva v6.1.3 software (BD Biosciences). Monocytes were gated via forward and side scatter. Doublets were excluded using a FSC-height vs. FSC-width dot plot. To maximize homogeneity and representativeness of the analyzed cell population, events were gated for CD14^+^ viability-dye^−^ cells.

### Cytokine immunoassays

For cytokine measurements, supernatants were collected at 24 h incubation and stored at −80°C until analysis. Concentrations of human TNF-α, IL-1β, IL-8, IL-12p40, IL-10, and IL-1ra were measured in duplicate by means of a multi-analyte immunoassay using Luminex® bead technology and reagent kits from Merck Millipore (Merck group, Darmstadt, Germany). The lower detection limits of the assays were 0.98 pg/ml (TNF-α), 1.35 pg/ml (IL-1β), 3.17 pg/ml (IL-8), 2.42 pg/ml (IL-12p40), 1.23 pg/ml (IL-10), and 2.42 pg/ml (IL-1ra). A curve was fit to the standards using xPonent® Software (Luminex Cooperation, Austin, Texas, USA), and cytokine concentrations from each sample were calculated from the standard curve.

### Statistical analysis

Prism® 6 software (GraphPad Software, San Diego, CA, USA) was used for statistical analysis. Data are expressed as mean ± standard deviation (SD). Differences among groups were analyzed using the non-parametric Kruskal-Wallis test and Dunn's multiple comparison *post-hoc* test. Mann-Whitney *U*-test was used to compare stimulation intensities between corresponding neonatal and adult monocyte subsets. Statistical significance was defined as *p*-value < 0.05.

## Results

### Basal cytokine expression in cord blood and adult monocytes

**Figure 1:**
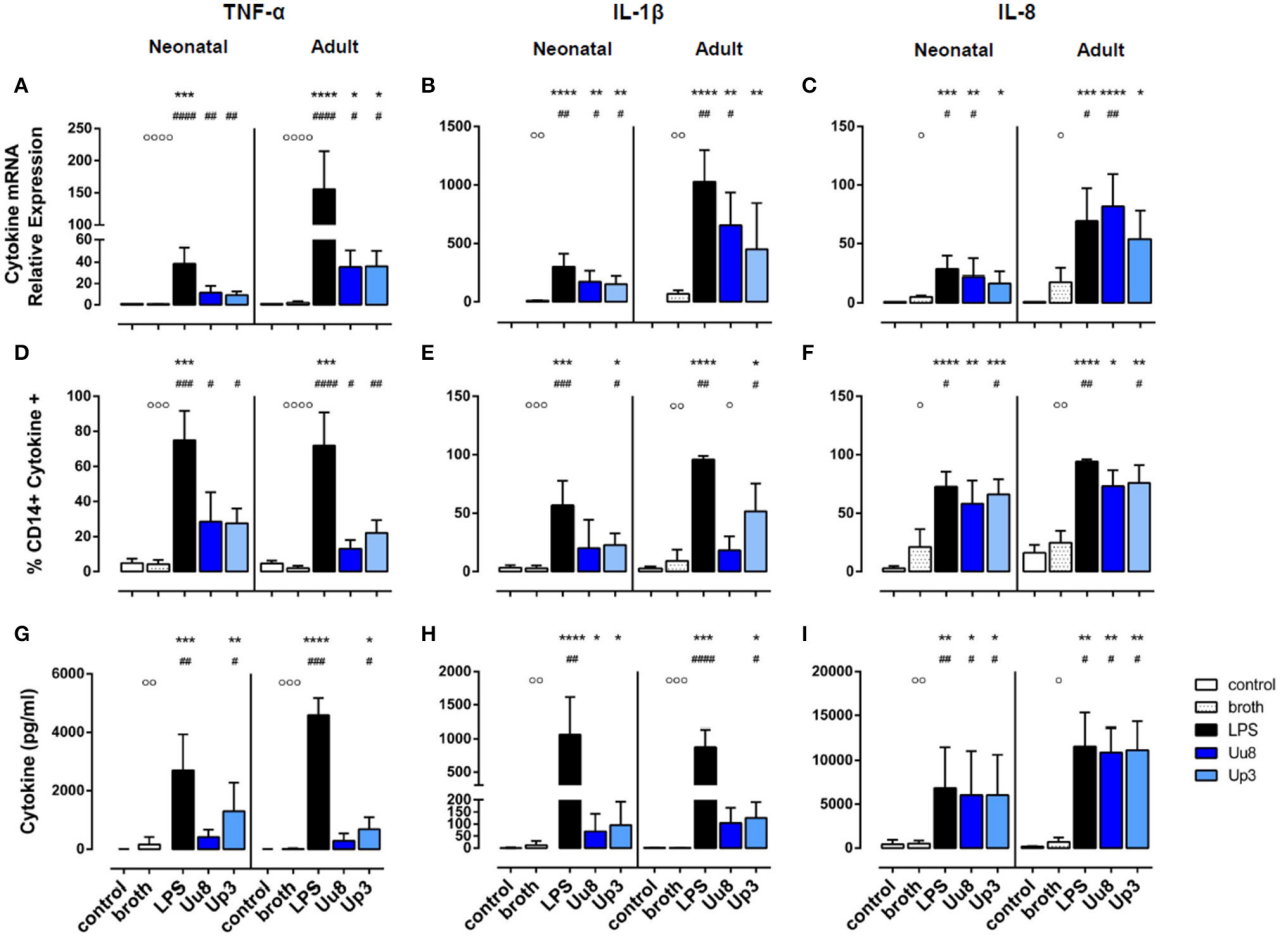
Pro-inflammatory TNF-α, IL-1β, and IL-8 responses in term neonatal and adult human monocytes exposed to *Ureaplasma* serovars Uu8 and Up3. Relative quantification of cytokine mRNA **(A–C)**, percentage of cytokine-positive CD14^+^ monocytes **(D–F)** and cytokine concentration in the supernatant **(G–I)** are presented as mean ± *SD*. Unstimulated cells and monocytes exposed to in-house *Ureaplasma* medium (broth) served as negative controls. Monocytes stimulated with *E. coli* LPS served as positive control (^*^p < 0.05, ^**^p < 0.01, ^***^p < 0.001, ^****^p < 0.0001, vs. unstimulated control; ^#^*p* < 0.05, ^##^*p* < 0.01, ^###^*p* < 0.001, ^####^*p* < 0.0001, vs. broth control; °*p* < 0.05, ^°°^*p* < 0.01, ^°°°^*p* < 0.001, ^°°°°^*p* < 0.0001, vs. LPS-stimulated monocytes).

**Figure 2:**
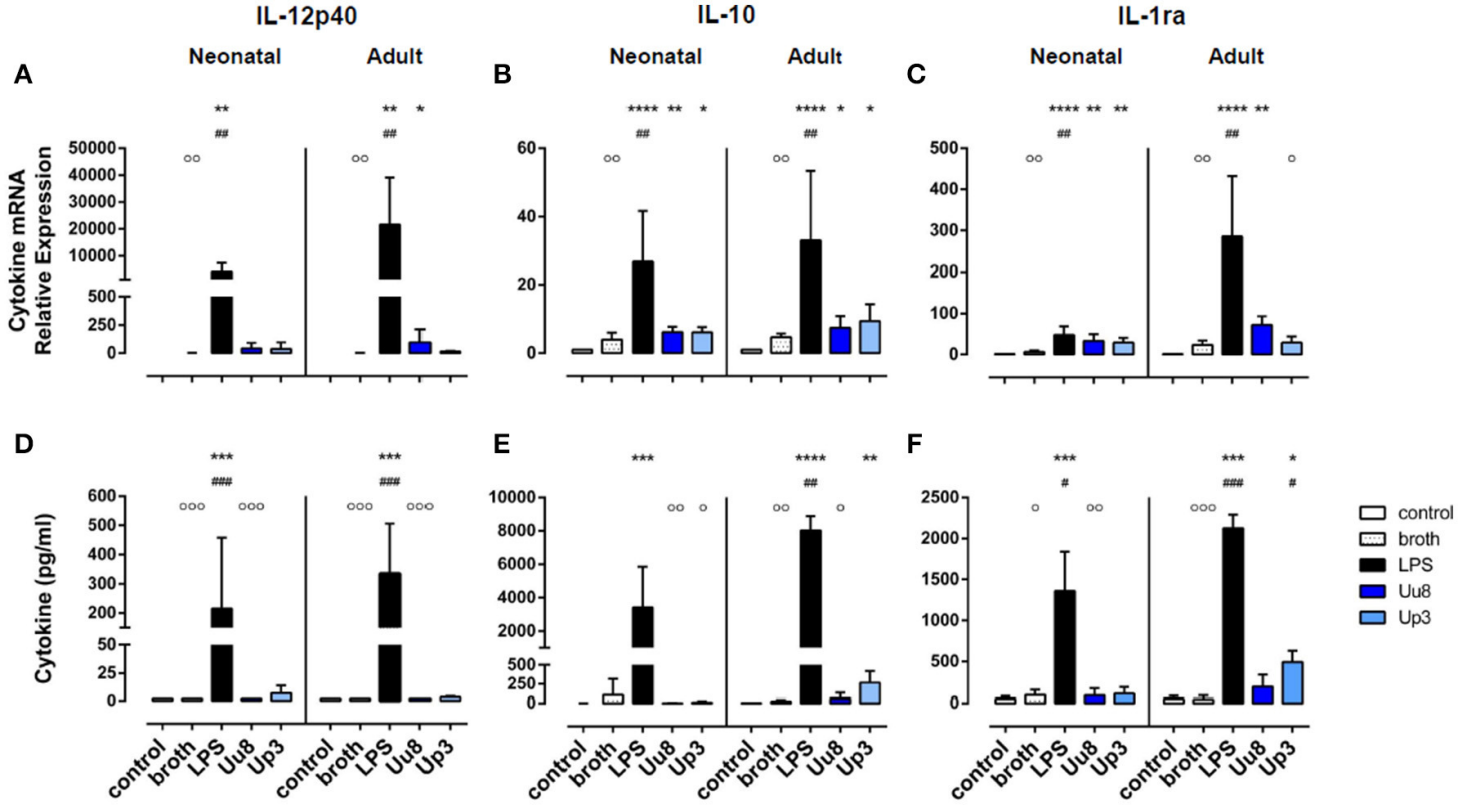
Immunoregulatory IL-12p40 and anti-inflammatory IL-10 and IL-1ra responses in *Ureaplasma*-stimulated term neonatal and adult monocytes. Cytokine expression was assessed at the level of mRNA **(A–C)** and protein secretion **(D–F)**. The values represent the means ± *SD* (^*^p < 0.05, ^**^p < 0.01, ^***^p < 0.001, ^****^p < 0.0001, vs. unstimulated control; ^#^*p* < 0.05, ^##^*p* < 0.01, ^###^*p* < 0.001, vs. broth control; °*p* < 0.05, ^°°^*p* < 0.01, ^°°°^*p* < 0.001, vs. LPS-stimulated monocytes).

In the absence of any stimulus, both term neonatal and adult monocytes exhibited negligible cytokine expression (Figures 1, 2). Since pathogen-induced immune responses may be regulated at the level of transcription and translation, we correlated mRNA expression with protein secretion using qRT-PCR and multi-analyte immunoassay. For TNF-α, IL-1β, IL-8, and IL-10, we quantified corresponding intracellular protein synthesis by means of polychromatic flow cytometry. Due to a limitation of fluorochromes, we cannot provide data on intracellular expression of IL-12 and IL-1ra. Evaluation of intracellular IL-10 synthesis was hampered by rare events both upon stimulation with ureaplasmas and LPS (data not shown).

Upon stimulation with *E. coli* LPS, we detected profound pro- and anti-inflammatory cytokine responses in both monocyte subsets in terms of mRNA and protein expression (Figures 1, 2). However, compared to adult monocytes, LPS-stimulated cord blood monocytes showed less pronounced mRNA expression of TNF-α, IL-1β and IL-8 (*p* < 0.001 each), displayed lower intracellular synthesis of IL-1β (*p* = 0.001) and released lower amounts of TNF-α (*p* = 0.026) and IL-8 (*p* = 0.049). Moreover, cord blood monocytes displayed lower levels of LPS-induced IL-12p40 mRNA (*p* = 0.032, vs. adult monocytes) and LPS-induced IL-10 release (*p* = 0.002) and exerted less pronounced IL-1ra responses upon LPS stimulation, both at the level of mRNA (*p* = 0.001) and protein expression (*p* = 0.026).

### Pro-inflammatory cytokine responses in *Ureaplasma*-stimulated monocytes

In term neonatal monocytes, Uu8 and Up3 significantly increased mRNA expression of TNF-α (Uu8: *p* = 0.004, Up3: *p* = 0.008) and IL-1β mRNA (Uu8: *p* = 0.040, Up3: *p* = 0.046) compared to broth control (Figures 1A,B). Induction of IL-8 mRNA was significant for Uu8 (Uu8: *p* = 0.044) (Figure 1C). Both isolates enhanced corresponding synthesis of intracellular TNF-α (Uu8: *p* = 0.049, Up3: *p* = 0.041, vs. broth control), and Up3 also increased expression of intracellular IL-1β and IL-8 (IL-1β: *p* = 0.021, IL-8: *p* = 0.043). Moreover, Up3 significantly enhanced levels of secreted TNF-α (*p* = 0.02), and both isolates stimulated secretion of IL-8 (Uu8: *p* = 0.048, Up3: *p* = 0.019) in neonatal monocytes (Figures 1G,I). Of note, these results were more significant when compared to unstimulated monocytes (Figures 1B,C,F–H). However, to exclude confounding effects of *Ureaplasma* medium (broth), *Ureaplasma* effects were compared to broth control throughout the study. The stimulatory effect of Uu8 and Up3 on TNF-α, IL-1β, and IL-8 responses was dose-dependent, as determined in preliminary stimulation assays (Supplementary Figure 1, data given for TNF-α, IL-1β, and IL-8 mRNA). Comparing the two isolates, we found no differences in terms of their pro-stimulatory capacity. Although *Ureaplasma*-induced neonatal cytokines appeared to be less pronounced than LPS-induced cytokine expression, those differences did not reach statistical significance.

In adult monocytes, both *Ureaplasma* isolates significantly induced TNF-α (Uu8: *p* = 0.027, Up3: *p* = 0.020, vs. broth control), and Uu8 also increased IL-1β and IL-8 mRNA (IL-1β: *p* = 0.037, IL-8: *p* = 0.0079) (Figures 1A–C). Both isolates enhanced intracellular protein synthesis of TNF-α (Uu8: *p* = 0.049, Up3: *p* = 0.024), and exposure to Up3 additionally induced intracellular expression of IL-1β (*p* = 0.048) as well as IL-8 (*p* = 0.040) (Figures 1D–F). Secretion of TNF-α and IL-1β was significantly increased upon Up3-stimulation (TNF-α: *p* = 0.046, IL-1β: *p* = 0.017) (Figures 1G,H). In accordance to neonatal monocytes, the inflammatory response observed was proportional to the dose of Uu8 or Up3 administered (data not shown), and did not significantly vary from one isolate to the other. *Ureaplasma*-induced pro-inflammatory cytokine levels did not significantly differ from LPS-mediated levels except for lower levels of intracellular IL-1β in Uu8-stimulated adult monocytes (*p* = 0.034). For the most part, *Ureaplasma*-induced mRNA expression of TNF-α, IL-1β and IL-8 was less pronounced in term neonatal monocytes compared to adult cells (Up3-induced IL-8: *p* = 0.012, others *p* < 0.001 each). However, only in terms of Up3-induced IL-8, these differences were paralleled by significantly lower levels of protein release (*p* = 0.043, vs. adult cells).

### *Ureaplasma*-induced immunoregulatory and anti-inflammatory cytokine responses in human monocytes

We did not detect any induction of IL-12p40 mRNA or IL-12p40 protein either in Uu8 or Up3-stimulated neonatal cells (Figures 2A,D). This difference to LPS was, in part, statistically significant for IL-12p40 release (Uu8: *p* = 0.0003). Compared to broth control, moreover, neither Uu8 nor Up3 induced mRNA and protein expression of IL-10 and IL-1ra (Figures 2B,C,E,F). This lack of cytokine induction in *Ureaplasma*-exposed monocytes differed significantly from LPS-stimulated neonatal cells (IL-10, Uu8: *p* = 0.002, Up3: *p* = 0.034; IL-1ra, Uu8: *p* = 0.009).

In accordance to neonatal cells, we found no induction of IL-12p40 and IL-10 in *Ureaplasma*-stimulated adult monocytes, neither at the level of mRNA nor protein expression compared to broth control (Figures 2A–D). Only Up3-induced IL-1ra release reached statistical significance (*p* = 0.013). Compared to LPS-induced cytokine levels, protein expression of IL-12p40 and IL-10 was significantly less pronounced in Uu8-stimulated adult monocytes (IL-12p40: *p* = 0.0003; IL-10: *p* = 0.038). Again, the two isolates did not significantly differ in terms of cytokine induction. Comparing term neonatal and adult monocytes, we found similar mRNA expression of IL-10 but higher levels of secreted IL-10 in *Ureaplasma*-stimulated adult cells (*p* = 0.002 for both isolates). Moreover, *Ureaplasma*-exposed adult monocytes showed higher levels of IL-1ra mRNA (*p* = 0.004) and a stronger release of IL-1ra protein upon Up3-exposure compared to neonatal cells (*p* = 0.002).

### Cytokine imbalances provoked by *Ureaplasma* species

Given the low expression of neonatal and adult IL-10 in *Ureaplasma*-exposed monocytes, cytokine ratios of IL-1β/IL-10 and IL-8/IL-10 were significantly more in favor of pro-inflammation than the corresponding ratios provoked by *E. coli* LPS (*p* < 0.01 and *p* < 0.001) (Table 2). These results were assessed in both monocytes subsets and were paralleled by significantly higher ratios of IL-8/IL-1ra protein in neonatal and adult monocytes (*p* < 0.01 and *p* < 0.001). In cord blood cells, this tendency toward cytokine imbalance was observed for both *Ureaplasma* isolates. In adult monocytes, the effect was primarily observed in Uu8-stimulated cells. However, except for the ratios of IL-1β/IL-10 mRNA (*p* < 0.01), TNF-α/IL-1ra mRNA (*p* < 0.05) and TNF-α/IL-10 protein (*p* < 0.05), differences among both isolates were not statistically significant.

**Table 2:** Ratios of pro-inflammatory to anti-inflammatory cytokines calculated at the level of mRNA and protein expression.

**Cytokine ratio**	**Neonatal monocytes**			**Adult monocytes**		
	**LPS**	**Uu8**	**Up3**	**LPS**	**Uu8**	**Up3**
**mRNA**						
TNF-α/IL-10	1.82 ± 1.01	1.92 ± 0.95	1.49 ± 0.41	7.66 ± 7.36	6.02 ± 3.90	4.85 ± 3.47
IL-1β/IL-10	16.91 ± 12.45	30.17 ± 17.73	26.13 ± 11.45	38.52 ± 14.79	94.15 ± 41.42^##^^,^^••^	43.23 ± 18.27
IL-8/IL-10	1.23 ± 0.82	3.38 ± 1.88^##^	2.66 ± 1.27	2.73 ± 1.64	12.48 ± 6.10^###^	5.91 ± 0.92
TNF- α/IL-1ra	1.17 ± 1.30	0.38 ± 0.21	0.37 ± 0.26	1.64 ± 0.33	0.53 ± 0.31^•^	1.70 ± 1.36
IL-1β/IL-1ra	6.86 ± 3.48	5.34 ± 2.54	5.70 ± 2.50	4.50 ± 3.11	10.38 ± 5.08	19.45 ± 13.44
IL-8/IL-1ra	0.73 ± 0.48	0.75 ± 0.43	0.63 ± 0.25	0.67 ± 0.18	1.31 ± 0.70^#^	2.42 ± 1.48^#^
**RELEASED PROTEIN**						
TNF- α/IL-10	1.44 ± 1.68	2.33 ± 2.53^•^	117.60 ± 93.41^##^	0.58 ± 0.08	9.17 ± 9.19^#^	5.19 ± 5.98
IL-1β/IL-10	0.72 ± 0.81	15.16 ± 14.92^##^	14.05 ± 22.26^#^	0.11 ± 0.03	3.07 ± 2.83^###^	0.51 ± 0.26
IL-8/IL-10	2.9 ± 1.79	1746.7 ± 1043.1^##^	1364.2 ± 1388.8^#^	1.48 ± 0.57	343.6 ± 218.4^###^	55.48 ± 33.86
TNF- α/IL-1ra	2.24 ± 1.30	8.23 ± 9.04	7.62 ± 6.66^#^	2.18 ± 0.39	2.33 ± 2.53	1.90 ± 1.55
IL-1β/IL-1ra	0.99 ± 0.62	0.68 ± 0.37	0.72 ± 0.55	0.41 ± 0.11	1.29 ± 0.93	0.33 ± 0.20
IL-8/IL-1ra	4.92 ± 1.83	85.71 ± 39.96^##^	59.79 ± 20.95^#^	5.47 ± 1.83	114.16 ± 63.29^###^	28.62 ± 10.58

# *p < 0.05*,

## *p < 0.01*,

### p < 0.001, vs. LPS-activated monocytes;

• *p < 0.05*,

•• *p < 0.01, vs. Up3-stimulated monocytes*.

*Results are reported as mean ± SD. Data were analyzed by ANOVA and the Kruskai-Wallis test. Multiple comparisons were made using the Dunn's post-hoc test*.

### Differential modulation of LPS-induced cytokine responses by *Ureaplasma* isolates

**Figure 3:**
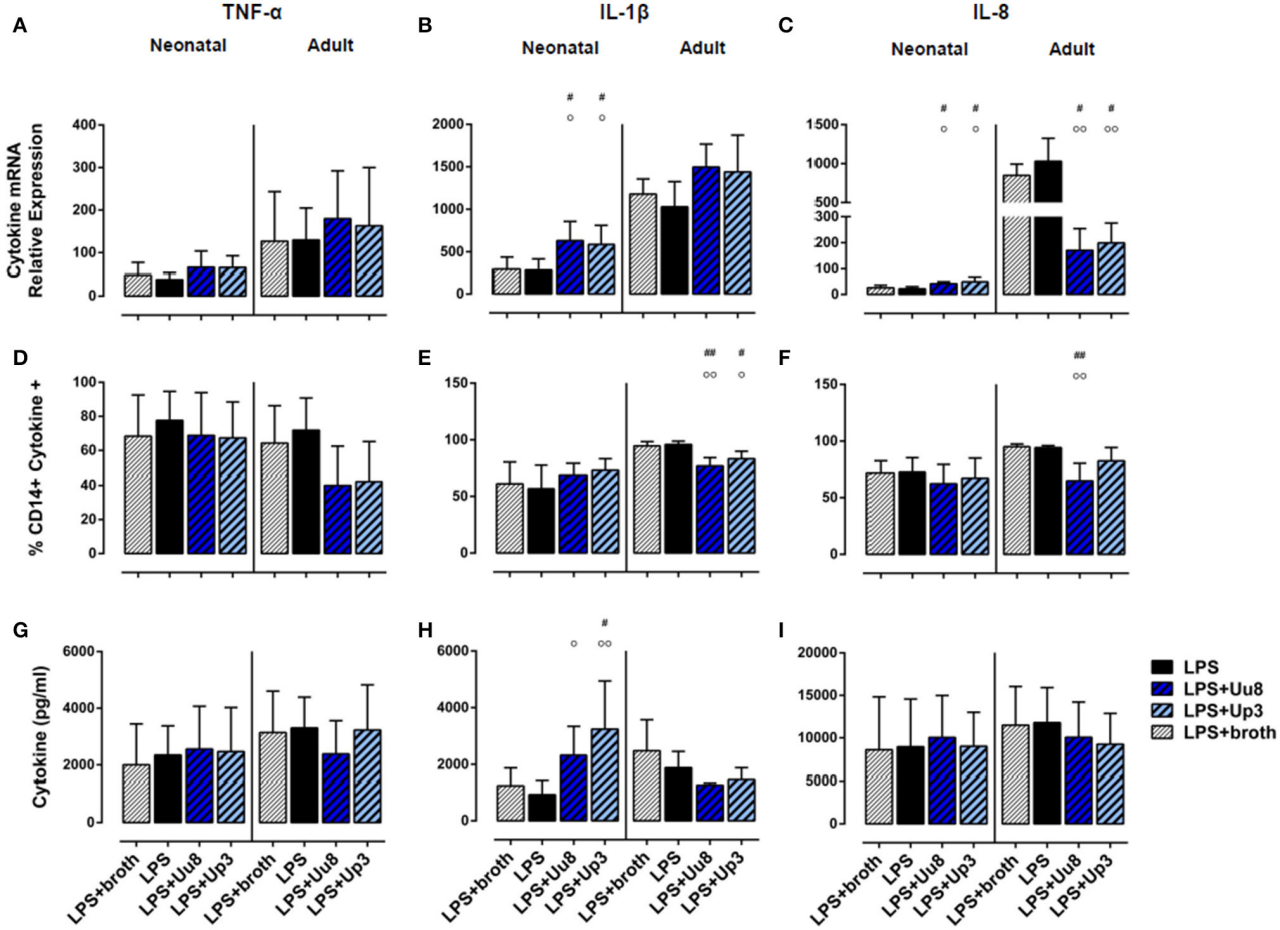
Modulation of pro-inflammatory cytokine responses in co-stimulated term neonatal and adult monocytes. Using qRT-PCR, cytokine flow cytometry and multi-analyte immunoassay, we assessed mRNA expression **(A–C)**, intracellular protein synthesis **(D–F)** as well as protein secretion **(G–I)** of each cytokine. LPS-activated cells served as positive control, LPS-primed monocytes exposed to *Ureaplasma* broth served as negative control. Values represent the means ± *SD* (^#^*p* < 0.05, ^##^*p* < 0.01, vs. broth control; °*p* < 0.05, ^°°^*p* < 0.01, vs. LPS-activated monocytes).

**Figure 4:**
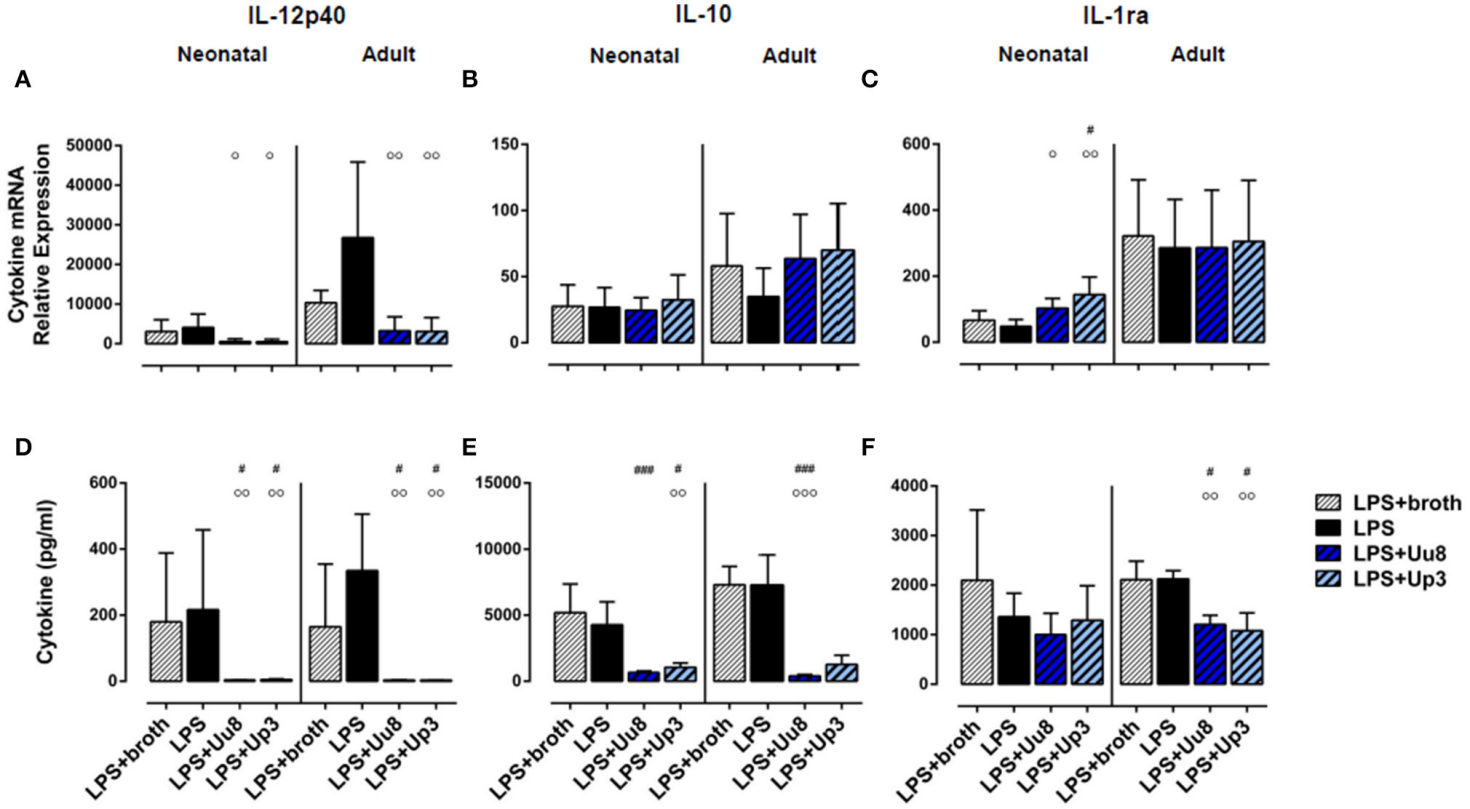
IL-12p40, IL-10 and IL-1ra responses in term neonatal and adult monocytes co-stimulated with Uu8 or Up3 and *E. coli* LPS. Relative quantification of cytokine mRNA **(A–C)** and concentration of secreted protein **(D–F)** are presented as mean ± *SD* (^#^*p* < 0.05, ^###^*p* < 0.001, vs. broth control; °*p* < 0.05, ^°°^*p* < 0.01, ^°°°^*p* < 0.001, vs. LPS-activated monocytes).

In co-stimulated neonatal monocytes, Uu8 and Up3 both amplified LPS-induced mRNA expression of IL-1β (Uu8: *p* = 0.031, Up3: *p* = 0.048, vs. monocytes stimulated with LPS + broth) and IL-8 (Uu8: *p* = 0.027, Up3: *p* = 0.043), and enhanced IL-1β secretion (Up3: *p* = 0.045) (Figures 3B,C,H). Moreover, we found a reduction of LPS-induced neonatal IL-12p40 release (Uu8: *p* = 0.025, Up3: *p* = 0.039) (Figure 4D) and a suppression of LPS-induced IL-10 (Uu8: *p* = 0.0009, Up3: *p* = 0.016) (Figure 4E). Although Up3 slightly increased LPS-mediated IL-1ra mRNA expression (*p* = 0.028), levels of LPS-induced IL-1ra release were not affected by co-stimulation with *Ureaplasma* isolates (Figures 4C,F).

In LPS-primed adult monocytes, co-exposure to Uu8 and Up3 resulted in a suppression of LPS-mediated intracellular IL-1β synthesis (Uu8: *p* = 0.006, Up3: *p* = 0.048, vs. monocytes stimulated with LPS + broth) (Figure 3E) and a reduction of LPS-induced mRNA and protein expression of IL-8 (mRNA, Uu8: *p* = 0.021, Up3: *p* = 0.033; intracellular protein, Uu8: *p* = 0.007) (Figures 3C,F). Moreover, we found a reduction of LPS-induced IL-12p40 secretion (Uu8: *p* = 0.041, Up3: *p* = 0.026) and attenuated IL-1ra release in co-stimulated adult monocytes (Uu8: *p* = 0.027, Up3: *p* = 0.016) (Figures 4A,D,F). LPS-induced release of IL-10 was diminished upon co-exposure to Uu8 (*p* = 0.0009), while suppressive effects of co-stimulation with Up3 did not reach statistical significance (*p* = 0.080, vs. monocytes stimulated with LPS + broth) (Figure 4E).

### *Ureaplasma*-driven modification of TLR2 and TLR4 expression in human monocytes

**Figure 5:**
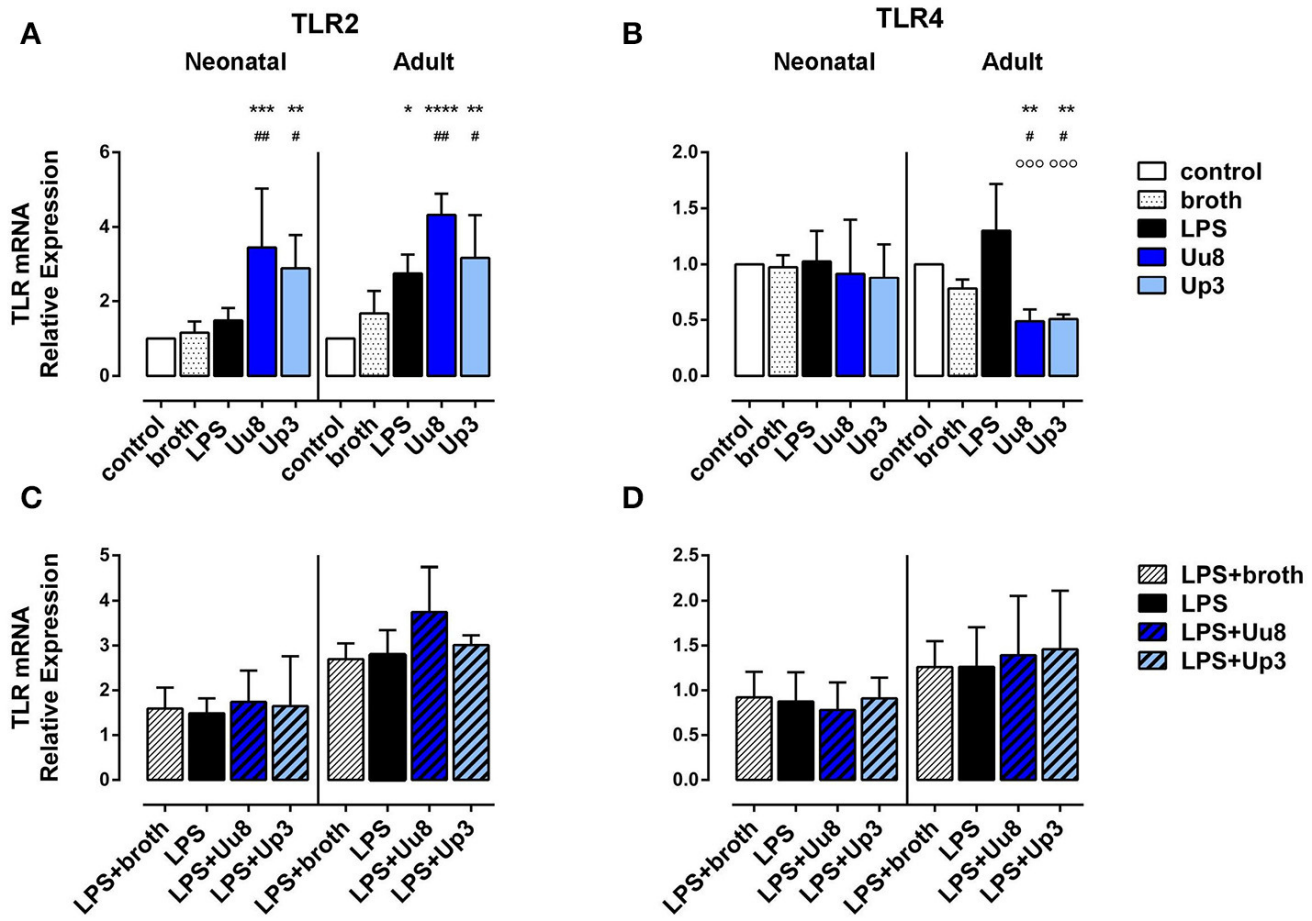
Expression of TLR2 and TLR4 mRNA in *Ureaplasma*-stimulated term neonatal and adult monocytes. Relative quantification is presented as mean ± *SD* in monocytes exposed to Uu8 and Up3 **(A,B)** and in monocytes co-stimulated with *Ureaplasma* isolates and LPS **(C,D)** (^*^p < 0.05, ^**^p < 0.01, ^***^p < 0.001, ^****^p < 0.0001, vs. unstimulated control; ^#^*p* < 0.05, ^##^*p* < 0.01, vs. broth control; ^°°°^*p* < 0.001, vs. LPS-activated monocytes).

Both isolates induced TLR2 mRNA in term neonatal (Uu8: *p* = 0.007, Up3: *p* = 0.011) and adult monocytes (Uu8: *p* = 0.003, Up3: *p* = 0.045) and suppressed TLR4 mRNA in adult cells (Uu8: *p* = 0.047, Up3: *p* = 0.039) (Figures 5A,B). Incubation of monocytes with *Ureaplasma* broth alone did not modify mRNA expression of TLR2 and TLR4. Corresponding surface expression of TLR2 and TLR4 protein was assessed by means of flow cytometry, documenting very low basal expression of TLR2 and TLR4 in both monocyte subsets at 14 h assessment time. The latter was neither affected by Uu8 nor by Up3 (data not shown). Contrary to *Ureaplasma* spp. stimulation with *E. coli* LPS did not modulate TLR2 or TLR4 expression either at the level of mRNA synthesis or outer membrane expression, except for LPS-induced adult TLR2 mRNA (*p* = 0.016) (Figure 5A). Co-stimulation with *Ureaplasma* isolates and the endotoxin did not modify TLR2 and TLR4 expression compared to LPS stimulation alone (Figures 5C,D, data shown for mRNA expression).

## Discussion

It is still debated controversial, whether *Ureaplasma* spp. are harmless commensals or driving force in the pathogenesis of intrauterine and neonatal infection (Steel et al., 2005; Volgmann et al., 2005; Glaser and Speer, 2015; Donders et al., 2017; Sweeney et al., 2017). In the current study, both *U. urealyticum* serovar 8 and *U. parvum* serovar 3 significantly induced TNF-α, IL-1β, and IL-8 responses in neonatal and adult monocytes. These results support previous findings from clinical studies, documenting increased expression of TNF-α, IL-1β, and IL-8 in airway secretions, serum specimen and amniotic fluid of preterm and term infants and preterm deliveries, respectively, upon *Ureaplasma* colonization and/or infection (Groneck et al., 1996; Patterson et al., 1998; Yoon et al., 1998; Viscardi et al., 2004; Goldenberg et al., 2008; Viscardi and Hasday, 2009; Kasper et al., 2010; Oh et al., 2010; Kacerovsky et al., 2012). Animal models of intrauterine infection with *Ureaplasma* spp. in mice, sheep and rhesus macaques, documented increased expression of pro-inflammatory cytokines in different tissues and variable intensities of inflammation (Yoder et al., 2003; Normann et al., 2009; Collins et al., 2010; Von Chamier et al., 2012; Senthamaraikannan et al., 2016). Few previous *in vitro* studies in monocytes and macrophages documented pro-inflammatory TNF-α, IL-6 and IL-8 responses upon stimulation with individual *Ureaplasma* strains (Manimtim et al., 2001; Li et al., 2002a,b). Monocyte-derived TNF-α, IL-1β and IL-8 are key mediators of early inflammation, orchestrating a cascade of cellular and humoral responses (Arango Duque and Descoteaux, 2014). With infection-related preterm labor resulting from a production of pro-inflammatory mediators (Wenstrom et al., 1996; Romero et al., 2007), increased levels of TNF-α, IL-1β, and IL-8 may link intrauterine *Ureaplasma* infection with preterm birth. In neonates, prenatal, perinatal and postnatal *Ureaplasma* infection might contribute to or initiate a considerable injurious inflammatory response. Consecutive exaggerated TNF-α, IL-1β, and IL-8 expression has been repetitively associated with adverse pulmonary and neurodevelopmental outcome (Viscardi et al., 2004; Volpe, 2008; Speer, 2009; Dammann and Leviton, 2014).

Physiologically, inflammation is tightly controlled by a number of immunoregulatory cytokines, such as IL-12, or anti-inflammatory IL-10, and IL-1ra (Trinchieri, 2003; Sabat et al., 2010; Sims and Smith, 2010). In contrast to LPS, however, Uu8 and Up3 did not induce IL-12p40 responses either in neonatal or in adult monocytes. This heterodimeric cytokine, bridging innate and adaptive immune responses, is known to be an important mediator in bacterial infections with intracellular and extracellular bacteria (Mancuso et al., 1997; Trinchieri, 2003). Expression of the subunit *p40* seems to correlate with the synthesis of biologically active IL-12 and, moreover, may exert pro-inflammatory and immune-stimulatory functions itself (Abdi, 2002). IL-12 has not been studied in the context of *Ureaplasma* infection so far. However, in view of the potential invasion of host cells by *Ureaplasma* spp. (Waites et al., 2009; Nishiumi et al., 2017), our findings of missing IL-12 responses in Uu8 and Up3-stimulated monocytes might be of particular importance, potentially facilitating evasion of adaptive immune responses by the pathogen (Jayakumar et al., 2008).

Both *Ureaplasma* isolates induced negligible and significantly lower levels of anti-inflammatory IL-10 than *E. coli* LPS. These results confirm previous data from Viscardi and coworkers, who found lower IL-10 expression in *U. urealyticum*-stimulated preterm, term and adult monocytes compared to the corresponding LPS-activated monocyte subsets (Manimtim et al., 2001). IL-10 has been assigned a key role in the limitation of adverse inflammation, inhibiting the release of pro-inflammatory mediators, such as TNF-α, IL-1β, IL-8, and IL-12, and enhancing the release of anti-inflammatory mediators from monocytes and macrophages (Sabat et al., 2010). Impaired IL-10 responses upon *Ureaplasma* infection, thus, might predispose to exaggerated or prolonged inflammation. Of note, levels of IL-10 were diminished in bronchoalveolar lavage samples of preterm infants with BPD compared to term infants with respiratory failure, indicating a role for insufficient IL-10 expression in the pathogenesis of prolonged lung inflammation and chronic lung injury (Jones et al., 1996). The impact of bacterial colonization and/or infection has not been examined in this context so far. Our current findings suggest a role for *Ureaplasma* spp. in the observed cytokine imbalance.

Given the low amounts of *Ureaplasma*-induced anti-inflammatory IL-10 in neonatal and adult monocytes, ratios of pro-inflammatory cytokines to IL-10 were significantly more in favor of pro-inflammation than the corresponding ratios provoked by *E. coli* LPS. In terms of protein release, those effects were particularly obvious in term neonatal monocytes. Of note, inadequate IL-10 responses were correlated with poor outcome in systemic inflammation and adult respiratory distress syndrome (Donnelly et al., 1996). Exogenous administration of IL-10, on the contrary, attenuated pulmonary inflammation in animal models and in *in vitro* studies (Kwong et al., 1998; Mesples et al., 2003).

The current data further suggest a role of diminished IL-1ra expression in the pathogenesis of *Ureaplasma*-driven inflammation. Uu8 and Up3 induced significantly lower amounts of anti-inflammatory IL-1ra compared to *E. coli* LPS, both at the level of mRNA expression and protein secretion. Moreover, *Ureaplasma*-promoted cytokine ratios of pro-inflammatory IL-8 to anti-inflammatory IL-1ra were significantly more in favor of pro-inflammation than the corresponding ratios provoked by LPS. The counter-regulatory cytokine IL-1ra, a relevant antagonist of pro-inflammatory IL-1 signaling, has not been subject to *in vitro* investigations with *Ureaplasma* spp. so far. However, our findings are consistent with only modest increases in pulmonary IL-1ra expression in a sheep model of *Ureaplasma*-induced fetal inflammation, diminished expression of the counter-regulatory cytokine IL-6 in *U. urealyticum*-stimulated preterm, term and adult monocytes, and the clinical findings of increased levels of TNF-α and IL-8 but rather low levels of counter-regulatory cytokines in preterm infants with *Ureaplasma* respiratory tract colonization (Patterson et al., 1998; Manimtim et al., 2001; Kallapur et al., 2011). Both inadequate and imbalanced IL-1ra expression appears to predispose to severe neonatal and adult lung injury (Donnelly et al., 1996; Kakkera et al., 2005). In animal models of neonatal lung and brain injury, treatment with exogenous IL-1ra significantly reduced inflammation and inflammation-induced organ-injury (Nold et al., 2013; Nadeau-Vallee et al., 2017).

Upon co-stimulation, Uu8 and Up3 suppressed LPS-induced intracellular IL-1β and IL-8 in adult monocytes, while LPS-induced pro-inflammation was maintained and partly aggravated in neonatal cells. These data may point to immunomodulatory features of *Ureaplasma*. Since co-stimulated monocytes had been pre-incubated with LPS, the observed effects may not be attributed to a physical interference of *Ureaplasma* isolates with LPS. Consistent with our findings, *U. urealyticum* potentiated LPS-induced cytokine responses in a dose-dependent manner in previous co-exposure experiments with neonatal monocytes (Manimtim et al., 2001). Moreover, cytokine responses in fetal blood and lung monocytes following 7d exposure to *Ureaplasma* CA and additional 2d exposure to LPS significantly exceeded LPS-mediated levels in a sheep model (Kallapur et al., 2011). However, in the same model, chronic (70d) *Ureaplasma-*induced CA mitigated fetal immune responses to a second LPS stimulus (Kallapur et al., 2011). Our data may be of clinical importance, since CA is often related to polymicrobial infection (Kirchner et al., 2007; Digiulio et al., 2008). In concert with additional factors, such as the duration of infection, pathogen interaction, and host immune function, *Ureaplasma* infection might affect immune defense to a second pathogen, modify and aggravate inflammation and promote inflammatory organ injury. In neonates, *Ureaplasma* colonization and/or infection may also modulate neonatal outcome in conjunction with non-infectious harmful events. In preterm infants, prolonged ventilation additive to colonization with *Ureaplasma* spp. (but not with other bacteria) conferred an increased risk of severe chronic lung injury (Inatomi et al., 2012).

Particularities in the ontogeny of early life immune responses are thought to place term and especially preterm infants at high risk of severe infection and inflammation-related organ injury (Cuenca et al., 2013; Dowling and Levy, 2014). In fact, there is conflicting data on the responsiveness of pathogen-stimulated neonatal monocytes and their ability to mount appropriate pro-inflammatory responses. A number of studies point to a gestational age-dependent increase in pro-inflammatory and immunoregulatory cytokine responses in stimulated neonatal PBMCs (Forster-Waldl et al., 2005; Yerkovich et al., 2007; Lavoie et al., 2010). Comparing neonatal and adult monocyte cytokine responses in the current study, for the most part, we found less pronounced mRNA expression in LPS- and *Ureaplasma*-stimulated cord blood monocytes. However, only in LPS-stimulated monocytes, these differences were paralleled by smaller amounts of corresponding protein synthesis in neonatal cells. Levels of *Ureaplasma*-induced protein secretion did not significantly differ among cord blood and adult monocytes, with exception of lower Up3-induced IL-8 and IL-1ra in neonatal cells, indicating a similar pro-inflammatory capacity of *Ureaplasma* spp. in term neonatal and adult human monocytes. Of note, we had excluded cord blood donors with either a history of CA or clinical and laboratory evidence of such prior to the stimulation assays, in order to largely rule out confounding effects of antenatal exposures potentially affecting functional measurements of immune responses (Kramer et al., 2005; Netea et al., 2011).

Our data indicate immunomodulatory capacities of Uu8 and Up3 with respect to TLR2 and TLR4 expression. In contrast to LPS, Uu8 and Up3 significantly induced TLR2 mRNA in term neonatal and adult cells and suppressed TLR4 mRNA in adult monocytes. These findings may have clinical implication, since alterations in TLR2 and TLR4 expression may render monocytes particularly alert for invading pathogens and may increase the individual's susceptibility to severe infection (Williams et al., 2003; Lavoie et al., 2012; Glaser and Speer, 2013). Our data are, in part, in accordance with previous findings from animal models. Enhanced placental TLR2 expression was documented in a mouse model of *U. parvum*-induced CA (Allam et al., 2014). Increased mRNA levels of TLR1, TLR2, TLR4, TLR6, and TLR9 were found in the fetal ovine gut following *U. parvum* exposure (Wolfs et al., 2013). Data from epidemiologic and experimental studies indicate the contribution of TLR1, TLR2, TLR4, TLR6, and TLR9 signaling in *Ureaplasma*-mediated inflammation (Peltier et al., 2007; Shimizu et al., 2008; Triantafilou et al., 2013).

Little is known about differences in virulence among certain serovars (Abele-Horn et al., 1997; Sung et al., 2011; Paralanov et al., 2012; Sweeney et al., 2017). While some studies reported on a predominance of *U. parvum* in clinical isolates (Sung et al., 2011; Payne et al., 2012; Friedland et al., 2015), other studies failed to designate serovars more often associated with invasive disease and adverse pregnancy outcomes than others (Xiao et al., 2011; Paralanov et al., 2012). In the present study, we did not observe significant differences between *U. urealyticum* serovar 8 and *U. parvum* serovar 3 concerning the pro-inflammatory and immunomodulatory capacity. However, pre-selection of the serovars and the use of laboratory strains may have caused a selection bias. Most likely, pathogen virulence, host genetics and host immune factors as well as polymicrobial interaction and the duration of infection are critical determinants in the clinical course of *Ureaplasma* infection. Data from a mouse model of intrauterine infection with *U. parvum* suggest a strong impact of host genetic background on the clinical course of inflammation (Von Chamier et al., 2012; Allam et al., 2014).

*In vitro* data on *Ureaplasma*-driven innate immune responses are limited so far, not least on account of special demands of these pathogens on bacterial culture and the need of complex culture media. For reasons of confounding pro-inflammatory effects of some media, previous *in vitro* approaches and animal models often used heat-killed *Ureaplasma* and extracted or recombinant *Ureaplasma* outer membrane proteins (Li et al., 2002a; Peltier et al., 2007; Shimizu et al., 2008; Friedland et al., 2015). Having implemented the propagation of *Ureaplasma* isolates in yeast-free medium, the strength of this study relates to the use of viable bacteria. Moreover, the application of two distinct *Ureaplasma* serovars and the assessment of cytokine expression at the level of mRNA, intracellular protein synthesis and protein secretion separate the current approach from previous studies. However, some limitations ought to be acknowledged: Due to limited access to preterm cord blood to be further processed and limited numbers of purified preterm monocytes thereafter, we used monocytes from healthy term newborns in this first study. Future investigations ought to include preterm monocytes, in order to allow for a discrimination of *Ureaplasma*-induced cytokine responses among preterm and term or preterm and adult monocytes. Moreover, the net effect of pro- and anti-inflammatory cytokines *in vivo* may differ from *in vitro* conditions, since local and systemic factors, such as the timing and interaction of cytokine release, the surrounding immunological milieu, the presence of competing or amplifying elements or the responsiveness of target cells may exert varying influence (Arango Duque and Descoteaux, 2014; Dowling and Levy, 2014).

## Conclusion

Our data from acute stimulation assays demonstrate a considerable pro-inflammatory capacity of *Ureaplasma* spp. on monocyte-driven cytokine responses *in vitro*. Stimulating TNF-α, IL-1β and IL-8 expression, without inducing anti-inflammatory IL-10 and IL-1ra, *Ureaplasma* infection might push monocyte cytokine responses toward pro-inflammation. Moreover, the current study suggests immunomodulatory effects of *Ureaplasma* spp. comprising differential modification of monocyte-driven inflammation in the event of co-infection as well as alterations in TLR2 and TLR4 mRNA expression. Suppression of LPS-induced IL-1β and IL-8 in co-stimulated adult cells in contrast to sustained or aggravated neonatal inflammation may point to age-related immunomodulatory features of *Ureaplasma* spp. in the presence of a second stimulus. *Ureaplasma*-induced modulation of TLR2 and TLR4 mRNA might confer a heightened susceptibility to inflammatory signaling. We conclude that *Ureaplasma* infection may constitute monocyte-driven inflammation and consecutive morbidity. Both immune function and potential co-infections may be critical determinants in disease manifestation.

## Author contributions

All authors in the manuscript have significantly contributed. Study conception and design: KG, CS, BH, CS. Acquisition and analysis of data: KG, CS, MF, AW-G, HC. Interpretation of data: KG, CS, MF, AW-G, BH, HC, CS. Drafting and critical revision: KG, CS, MF, AW-G, BH, HC, CS. Final approval: KG, CS, MF, AW-G, BH, HC, CS. Agreement to be accountable for all aspects of the work: KG, CS, MF, AW-G, BH, HC, CS. No assistance was used in the preparation of the manuscript.

### Conflict of interest statement

The authors declare that the research was conducted in the absence of any commercial or financial relationships that could be construed as a potential conflict of interest.

## Supplementary material

Supplementary Figure 1 Preliminary dose-response and viability studies with *U. urealyticum* serovar 8, *U. parvum* serovar 3 and *E. coli* LPS (*n* = 3). Data are shown for term neonatal monocytes. Both isolates caused a dose-dependent induction of TNF-α, IL-1β and IL-8 mRNA expression at 4 h assessment **(A–C)** (^*^*p* < 0.05, ^**^*p* < 0.01, vs. unstimulated control; ^#^*p* < 0.05, ^##^*p* < 0.01, vs. 1 × 10^6^ CCU/ml; *p* < 0.05, vs. 1 × 10^7^ CCU/ml). Dose-dependent effects of LPS on TNF-α and IL-1β mRNA peaked at 100 ng/ml **(D–F)**. Stimulation neither with 100 ng/ml LPS nor exposure of monocytes to 1 × 10^8^ CCU/ml Uu8 and Up3 adversely affected cell viability. Representative dot plots of one experiment indicate fixable viability dye^+^ (apoptotic) cells in the upper left and right quadrants in un-stimulated neonatal monocytes and cells exposed to LPS, Uu8 or Up3 **(G)**.
