# Supplementary figures and images for: Ureaplasma Species Differentially Modulate Pro- and Anti-Inflammatory Cytokine Responses in Newborn and Adult Human Monocytes Pushing the State Toward Pro-Inflammation
Source: Front Cell Infect Microbiol. 2017 Nov 28;7:484. doi: 10.3389/fcimb.2017.00484 (PMC5712342; doi:10.3389/fcimb.2017.00484)

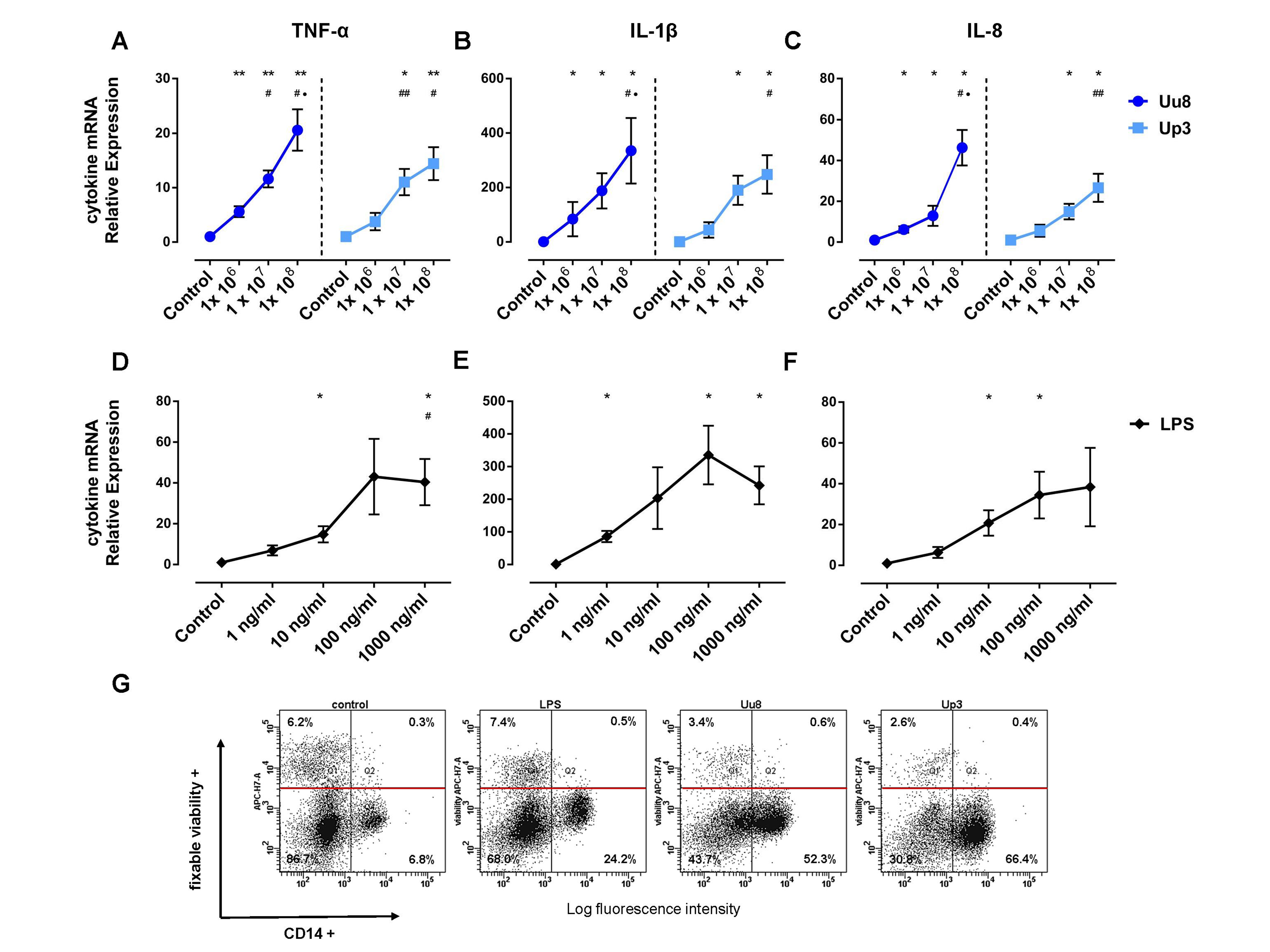

Supplement: Supplementary Figure 1 — Preliminary dose-response and viability studies with U. urealyticum serovar 8, U. parvum serovar 3 and E. coli LPS (n = 3). Data are shown for term neonatal monocytes. Both isolates caused a dose-dependent induction of TNF-α, IL-1β and IL-8 mRNA expression at 4 h assessment (A–C) (*p < 0.05, **p < 0.01, vs. unstimulated control; #p < 0.05, ##p < 0.01, vs. 1 × 10^6^ CCU/ml; p < 0.05, vs. 1 × 10^7^ CCU/ml). Dose-dependent effects of LPS on TNF-α and IL-1β mRNA peaked at 100 ng/ml (D–F). Stimulation neither with 100 ng/ml LPS nor exposure of monocytes to 1 × 10^8^ CCU/ml Uu8 and Up3 adversely affected cell viability. Representative dot plots of one experiment indicate fixable viability dye+ (apoptotic) cells in the upper left and right quadrants in un-stimulated neonatal monocytes and cells exposed to LPS, Uu8 or Up3 (G). [file Image1.TIF]
